# Management of a recurrent suicide attempt with needle insertion into the heart; a case report

**DOI:** 10.1016/j.ijscr.2025.112072

**Published:** 2025-10-16

**Authors:** Alireza Sadeghi, Nakisa Khansari, Seyed Kamaledin Hadei, Gholamreza Safarpour

**Affiliations:** a Student Research Committee, Hamadan University of Medical Sciences, Hamadan, Iran; b Department of Cardiology, School of Medicine, Hamadan University of Medical Sciences, Hamadan, Iran; c Department of Radiology, School of Medicine, Hamadan University of Medical Sciences, Hamadan, Iran; d Department of Heart Surgery, School of Medicine, Hamadan University of Medical Sciences, Hamadan, Iran

**Keywords:** Heart injuries, Suicide, Foreign bodies, Self-injurious behavior, Case reports

## Abstract

**Introduction:**

Penetrating cardiac injury (PCI) is rare and life-threatening, with complications that include hemorrhage, tamponade, arrhythmia, and cardiac arrest. Needle insertion into the heart is infrequently reported; to our knowledge, only one case of recurrent self-insertion has been described.

**Case presentation:**

A 29-year-old man with bipolar I disorder and a prior sternotomy for foreign-body removal presented after a second suicide attempt within two years, having self-inserted multiple needles through the chest and abdomen. Chest radiography showed three metallic densities in the left hemithorax and one in the abdominal wall. Transthoracic echocardiography demonstrated a linear metallic echo within the left ventricle (LV) near the apex. Computed tomography confirmed three metallic densities in the left thorax, one penetrating the LV to a depth of 30 mm. Median sternotomy was performed, and one needle was removed from the LV. After recovery, the patient was transferred to a psychiatric hospital for further inpatient treatment.

**Discussion:**

Intentional cardiac injury by needle insertion is extremely rare and poses diagnostic and therapeutic challenges. Early imaging and prompt surgery are essential to reduce morbidity and mortality. A multidisciplinary plan, including psychiatric evaluation and follow-up, is required to prevent fatal outcomes and recurrence.

**Conclusion:**

This study describes the successful management of a penetrating cardiac injury in a patient with a prior sternotomy.

## Highlights

• Penetrating cardiac trauma remains a rare but life-threatening injury.
• Self-insertion of needles into the heart is a medical and psychiatric emergency.
• Prompt diagnosis and intervention are needed to manage cardiac complications.
• A multi-disciplinary approach is needed to prevent recurrence.

## 1 Introduction

Penetrating cardiac injury (PCI) is rare and life-threatening, with complications that include cardiac tamponade, infection, embolism, valvular injury, arrhythmia, and cardiac arrest. Gunshot and stab wounds account for most cases. Other causes include blunt injury from fractured sternum or ribs and iatrogenic injury from needles, catheters, or trocars [1,2]. Intentional self-insertion of needles for self-harm has been reported only rarely and is usually associated with underlying psychiatric disorders [3]. Although isolated cases of intracardiac foreign bodies have been described, to our knowledge, only one case of recurrent self-insertion of needles has been reported [4].

Because of the high risk of lethal complications, prompt diagnosis and treatment are essential for survival in PCI [2]. Recurrent self-inflicted injury also suggests a severe underlying psychiatric disorder and requires a multidisciplinary approach to prevent further harm [5]. Here, we report a 29-year-old man with two similar suicide attempts involving multiple self-inserted foreign bodies into the chest wall and heart, who underwent two exploratory sternotomies within two years. This case report follows the SCARE checklist [6].

## 2 Case presentation

### 2.1 Background

A 29-year-old man was referred to the emergency department with the chief complaint of self-inserting sewing needles into his heart as a suicide attempt for the second time in two years. He had bipolar I disorder and had attempted a similar suicide 2 years earlier by self-inserting multiple foreign bodies through the chest and abdomen (Video 1). At that time, he underwent exploratory sternotomy, and all needles and foreign bodies were removed. After recovery, he was discharged with recommendations for regular follow-up in the cardiology and psychiatry outpatient clinics.

Past medical history included autosomal dominant polycystic kidney disease and hypertension. Medications were bisoprolol and VALSOMIX-HCT®, a fixed-dose combination of amlodipine, valsartan, and hydrochlorothiazide. He was also taking phenazopyridine. No psychiatric medication was recorded because he had discontinued psychiatric follow-up. He was a smoker and had a history of methadone use.

### 2.2 Investigations

**Fig. 1:**
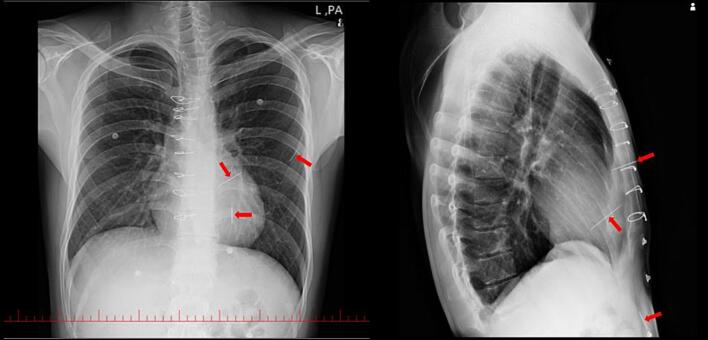
Frontal and lateral CXR show three linear metallic densities (red arrows) in the left hemithorax and one in the abdomen highly suggestive of foreign body such as needle.

**Fig. 2:**
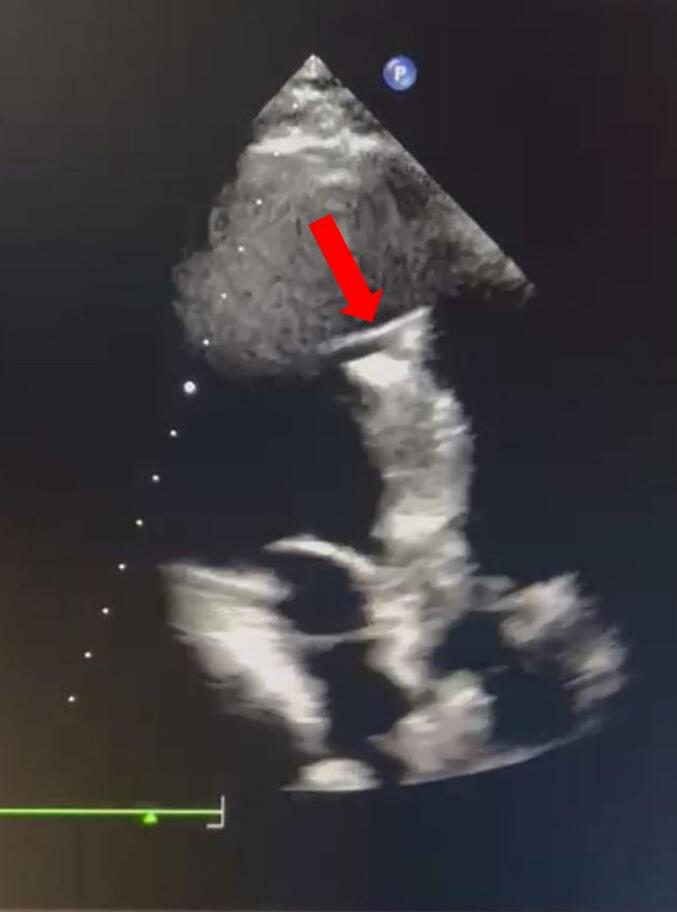
Apical three-chamber view of the 2D transthoracic echocardiogram. The red arrow shows the metallic shadow of the needle in the LV.

On admission, vital signs were stable. Chest radiography showed three metallic densities in the left hemithorax and one in the abdominal wall (Fig. 1). Transthoracic echocardiography demonstrated normal left ventricular (LV) size with mild systolic dysfunction (left ventricular ejection fraction [LVEF] 45 %), mild LV hypertrophy, and a metallic shadow within the LV (Fig. 2). The right ventricle (RV) was normal in size with mild systolic dysfunction. Valve assessment showed mild mitral regurgitation (MR), mild tricuspid regurgitation (TR), and mild pulmonary insufficiency. The aortic valve was bicuspid with mild-to-moderate aortic insufficiency (AI). A trivial pericardial effusion measuring 3 mm was noted inferior to the RV (Video 2).

**Fig. 3:**
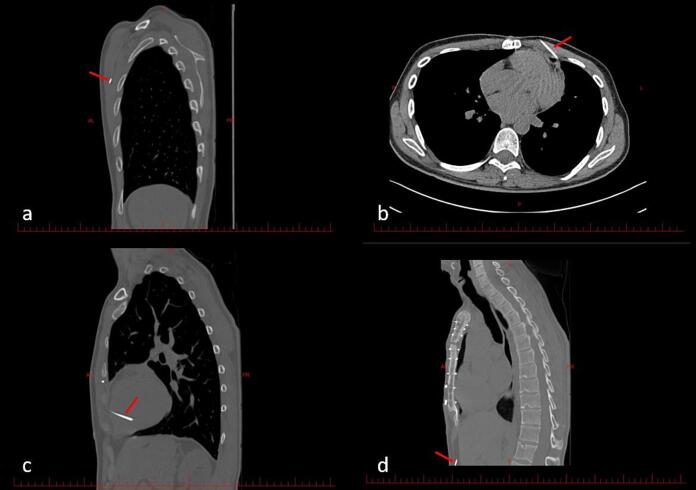
Axial and reformatted sagittal images of chest CT scan show a needle in the LV chamber (c) and three needles in the anterior thorax (a, b) and abdominal wall (d). red arrows show needles.

Following echocardiography, high-resolution computed tomography (HRCT) of the chest was performed. HRCT revealed three metallic densities in the left thoracic cavity, one of which penetrated the left ventricle (LV) to a depth of 30 mm. An additional needle was present in the abdominal wall (Fig. 3; Video 3). The sequence of events leading to presentation is summarized in Table 1.

### 2.3 Procedure

The patient was transferred to the operating room. After induction of general anesthesia, the chest was prepped and draped, and a median sternotomy was performed. Dense adhesions around the aorta and heart were released. A pericardiotomy was carried out, and traction sutures were placed. Intraoperatively, a 5 cm needle was identified traversing the left anterior thoracic wall and penetrating the left ventricle (LV). The needle was removed from the LV. Additional chest-wall needles could not be located. Hemostasis was achieved, and drains were placed. The aortic root was covered with pericardium. Recovery was uneventful, and the patient was transferred to the intensive care unit (ICU) with stable vital signs.

**Table 1:** The timeline.

Timeline	Event
December 2022	First suicidal attempt
December 2022	Moved to the cardiology hospital from the psychiatric hospital
December 2022	Six needles removed from the thorax
June 2024	Discontinuation of psychiatric drugs
7 January 2024, 2:06	Second suicidal attempt
7 January 2024, 9:29	Initial CXR
7 January 2024, 11:28	Echocardiography
7 January 2024, 13:58	Chest CT scan
10 January 2024	Second sternotomy, one needle removed from the heart
14 January 2024	moved to psychiatric hospital

### 2.4 Post-operative management and discharge

After the surgical removal of the needle penetrating the LV, the other two needles in the thoracic cavity and the one in the abdominal wall were managed conservatively. These needles were not surgically removed due to their stable position, absence of signs of acute complications such as infection or organ injury, and the increased risks associated with further surgical intervention, given the patient's condition and previous sternotomy.

**Fig. 4:**
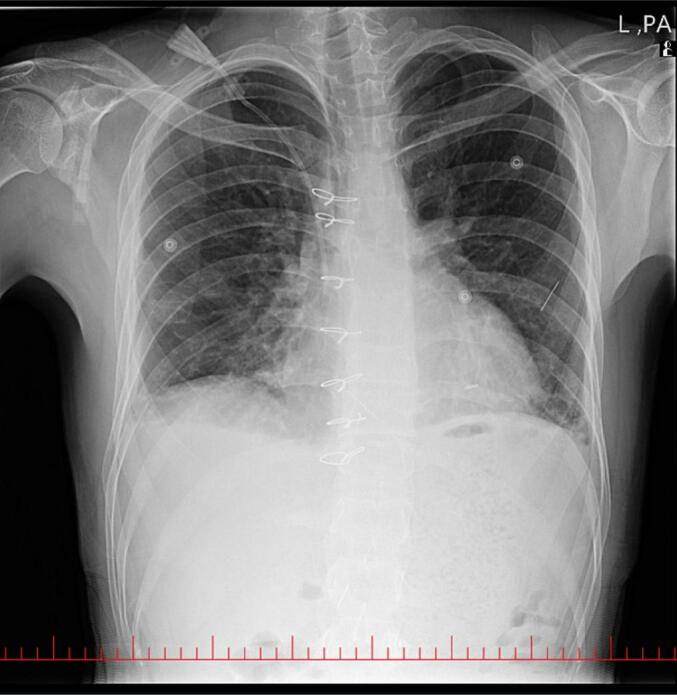
Post-operative frontal CXR after the removal of the needle from the LV.

Postoperative chest radiography showed no retained needle in the LV; the other two needles remained visible (Fig. 4). Transthoracic echocardiography on postoperative day 4 showed no intracardiac or pericardial foreign body, left ventricular ejection fraction (LVEF) 50 %, mild LV and RV systolic dysfunction, mild MR and TR, and mild-to-moderate AI. The patient was then transferred in good general condition to a psychiatric hospital for further evaluation and management.

## 3 Discussion

Needle penetration of the heart can occur in both children and adults. [7] and adults [3]. In pediatric cases, most cardiac injuries from needle penetration are accidental. In adults, intracardiac foreign bodies may result from accidental [8], iatrogenic [9], or intentional causes [3]. Reports describe intentional ingestion or self-insertion of needles for self-harm or suicide, most often in patients with underlying psychiatric disorders such as depressive disorders [10], schizophrenia [3], and substance abuse [11]. However, Intentional self-insertion into the chest without a diagnosed psychiatric disorder has also been reported [12].

In a recent study by Mohammadzadeh et al., 34 cases of self-insertion of needles in the English-language literature were reviewed. The majority of patients were female and required thoracotomy or sternotomy. In addition, the eventual outcome for most patients was improvement after therapeutic intervention [3]. To our knowledge, only one other case has been reported of recurrent self-inflicted cardiac injury by needles. She was a 17-year-old girl who inserted multiple needles into her chest two months after her first self-insertion of a needle into the right ventricle (RV) [4].

Although rare, PCI remains a challenging problem for surgeons. To prevent lethal complications and improve outcomes, clinicians need rapid evaluation and timely decision-making [2]. Initial assessment of a penetrating chest wound begins with a prompt, thorough history and physical examination. Injuries within the anatomic “cardiac box” carry the highest risk of cardiac injury. This region lies in the anterior thorax and is bordered superiorly by the clavicles, laterally by the nipples, and inferiorly by the xiphoid [13].

Chest radiography (CXR) is widely available and sensitive, and remains an indispensable first-line imaging study in the initial evaluation of penetrating chest trauma and suspected cardiac injury [14]. Transthoracic echocardiography (TTE) also plays a key role in early diagnosis and assessment by providing dynamic functional evaluation of the heart and direct visualization of pericardial effusion and intracardiac injuries [1,7]. Further evaluation with chest computed tomography (CT) can provide a detailed assessment in hemodynamically stable patients. Unstable patients should proceed to the operation immediately after resuscitation. In this case, the patient's hemodynamics were stable at admission, and a full clinical and paraclinical assessment was completed before surgery, including CXR, TTE, and high-resolution computed tomography (HRCT), which informed operative planning and increased the likelihood of a successful procedure.

No standard guidelines exist for managing retained intracardiac foreign bodies. In general, high-velocity objects frequently cause complications and should be removed, whereas low-velocity objects that are asymptomatic can often be managed non-operatively [7]. Operative options include median sternotomy, thoracotomy, or minimally invasive procedures, selected according to the object's location, size, depth of embedment, and the patient's hemodynamic stability [15]. The two principal incisions for cardiac injuries are median sternotomy and left lateral thoracotomy. Thoracotomy is preferred for patients who arrive in critical condition, while sternotomy is the incision of choice for hemodynamically stable patients [1]. Huang et al. reported video-assisted thoracoscopic surgery (VATS) removal of a sewing needle from the ventricular septum in a 32-year-old woman [12]. In all cases, the surgical approach and timing should be tailored to the patient's presentation and injury characteristics [1].

Given that a large needle had fully penetrated the left ventricle (LV) in this case, we chose to repeat median sternotomy to remove it. Median sternotomy provides superior exposure of the cardiac structures and allows cannulation for cardiopulmonary bypass if bleeding becomes uncontrollable [16]. It also permits a thorough inspection of the inner chest wall; by palpating externally, additional needles along a direct path to the heart or lungs can be identified and safely removed. The main risk of repeat sternotomy is direct injury to intrathoracic vessels, cardiac chambers, and lungs during sternal entry and adhesion dissection. Adhesions between 3 weeks and 6 months after the initial operation tend to be dense, inflammatory, and highly vascular, which increases the difficulty of dissection [17]. In this patient, the prior sternotomy had occurred about two years earlier, outside this window, so the risk of injury was lower.

In cases of intentionally inflicted cardiac injury as a suicide attempt, psychiatric concerns should be addressed alongside possible cardiac damage. Clinicians should adopt an empathic, supportive, and non-judgmental approach. A thorough psychiatric evaluation, including review of any history of psychiatric disorder and substance use, should be performed with a brief mental status examination, and the patient's coping mechanisms should be documented [18]. Predictors of recurrent self-harm include prior self-harm, hopelessness, history of psychiatric treatment, drug and alcohol misuse, schizophrenia, and living alone. Several tools assess the risk of self-harm and suicide, including the Manchester Self-Harm Rule, ReACT (Risk, Assessment, Crisis, Treatment) Rule, SAD PERSONS scale, and the Barratt Impulsiveness Scale (BIS). The Manchester Self-Harm Rule shows 97 % sensitivity for identifying patients who repeat self-harm within 6 months [19]. However, clinical guidelines in England, New Zealand, and Australia advise against using risk tools to predict suicide or repeated self-harm and recommend a comprehensive psychiatric assessment of individual needs and risk factors to guide management [20].

Our case demonstrates the importance of coordinated psychiatric care that involves health-care providers and the patient's family. The patient had bipolar disorder and a suicide attempt two years earlier; however, seven months before the second attempt, he was lost to psychiatric follow-up and had discontinued his medications, circumstances under which a more effective follow-up system might have prevented recurrence. Accordingly, strong medical planning and sustained emotional support are required in this and similar cases to reduce the risk of repeated self-harm and unfavorable outcomes. Such care should include structured aftercare, scheduled appointments, adherence monitoring, crisis-response planning, and facilitated access to substance-use services when indicated.

## 4 Conclusion

This case highlights the need for multidisciplinary management of intentionally self-inflicted cardiac injuries, with tailored surgical planning and early psychiatric intervention to minimize complications and prevent recurrence.

The following are the supplementary data related to this article.

Video 1 Chest CT scan of the patient after the first suicidal attempt shows metallic foreign bodies in the anterior thorax and pericardium. Video 1

Video 2 Echocardiography locates the metallic sewing needle in the LV chamber. Video 2

Video 3 CT scan of the chest revealing three needles in the thoracic cavity and one in the abdominal wall. Video 3

## List of abbreviations

PCI: Penetrating cardiac injury
CXR: Chest X-ray
LV: Left ventricle
LVEF: Left ventricular ejection fraction
RV: Right ventricle
MR: Mitral regurgitation
TR: Tricuspid regurgitation
AI: Aortic insufficiency
PE: Pericardial effusion
HRCT: High resolution computed tomography
CT: Computed tomography

## Consent

Written informed consent was obtained from the patient for publication of this case report and accompanying images. A copy of the consent is available for review by the Editor-in-Chief of this journal upon request.

## Ethical approval

The Ethics Committee of the faculty approved this study.

## Guarantor

Dr. Seyed Kamaledin Hadei accepts full responsibility for the work and approves the whole process from designing the study to publish.

## Funding

This research received no specific grant from public, commercial, or not-for-profit funding agencies.

## CRediT authorship contribution statement

**Alireza Sadeghi:** Project administration, Data curation, Investigation, Writing – original draft. **Nakisa Khansari:** Conceptualization, Data curation, Writing – review & editing. **Seyed Kamaledin Hadei:** Conceptualization, Supervision, Data curation, Writing – review & editing. **Gholamreza Safarpour:** Data curation, Writing – review & editing.

## Declaration of competing interest

The authors declare that they have no conflicts of interest.
